# Mixed Convective Peristaltic Flow of Water Based Nanofluids with Joule Heating and Convective Boundary Conditions

**DOI:** 10.1371/journal.pone.0153537

**Published:** 2016-04-22

**Authors:** Tasawar Hayat, Sadaf Nawaz, Ahmed Alsaedi, Maimona Rafiq

**Affiliations:** 1 Department of Mathematics, Quaid-I-Azam University 45320, Islamabad 44000, Pakistan; 2 Nonlinear Analysis and Applied Mathematics (NAAM) Research Group, Department of Mathematics, Faculty of Sciences, King Abdulaziz University, Jeddah 21589, Saudi Arabia; Tsinghua University, CHINA

## Abstract

Main objective of present study is to analyze the mixed convective peristaltic transport of water based nanofluids using five different nanoparticles i.e. (Al_2_O_3,_ CuO, Cu, Ag and TiO_2_). Two thermal conductivity models namely the Maxwell's and Hamilton-Crosser's are used in this study. Hall and Joule heating effects are also given consideration. Convection boundary conditions are employed. Furthermore, viscous dissipation and heat generation/absorption are used to model the energy equation. Problem is simplified by employing lubrication approach. System of equations are solved numerically. Influence of pertinent parameters on the velocity and temperature are discussed. Also the heat transfer rate at the wall is observed for considered five nanofluids using the two phase models via graphs.

## Introduction

Nanofluid is a type of fluid engineered by dispersing nanometer size materials e.g. nanoparticles, nanorods, nanotubes and so on in conventional fluids like water, ethylene-glycol and oil etc. Up to now, much more attention has been given to the potentials of nanofluids in practical applications among these heat transfer enhancement is the most significant issue. The term nanofluids was initially used by Choi [1]. The commonly used nanoparticles are metals (Cu, Ag, Fe, Au), metallic oxides (CuO, Al_2_O_3_, TiO_2_, ZnO), nitride/carbide ceramics (AlN, SiN, SiC, TiC), and carbon nanotubes etc. The most commonly used base fluids are water, ethylene-glycol and oil etc. Because of the property of enhancing the heat transfer rate the nanofluids are extensively used in automobiles as coolant. In welding equipments, nanofluids are used to cool high heat-flux devices such as high power microwave tubes and high power laser diode arrays. The measurement of nanofluids critical heat flux (CHF) in a forced convection loop is very useful for nuclear applications. Wide variety of industrial applications ranging from transportation to energy production, electronic systems like microprocessors, Micro-Electro-Mechanical Systems (MEMS) and biotechnology involves the use of nanofluids. Some of the investigations on the nanofluids are given through the references [2–10]. Several models are used to estimate the thermal conductivity of nanofluids. However, Maxwell's [11] and Hamilton Crosser's [12] models are extensively used.

Peristaltic mechanism is important in physiology for the transport of fluids. This mechanism is induced due to the sinusoidal wave along the walls which propel the fluid. It is extensively found in the human body for the transport of food through esophagus, transport of urine from kidneys to bladder, fluid mechanics in the perivascular space of the brain etc. Besides these it is used in industry for sanitary fluid transport. Many devices for example heart lung machine, hose pump, peristalsis pump etc, are operated under this principle. Transport of water to all branches of tree are due to the same principle. Due to these developments the peristalsis has become an important topic for research and some literature in this regard can be seen through the references [13–26].

Due to the advancement in medical science many diseases are cured by the use of colloidal drug delivery. In the drug delivery system with the help of magnetic fluxes the magnetic nanoparticles with the drug are sent to the tumor side. With the help of applied magnetic field it is possible to control the magnetic-nanoparticles in the human body towards the tumor site. Now a days in the modern drug delivery system the peristaltic transport of nanofluid has gained the attention. Some studies dealing with the peristaltic flows of nanofluids can be consulted through the studies [27–35].

In this study mixed convective peristaltic transport of water based nanofluids is considered. Influence of constant applied magnetic field in an asymmetric channel is taken into account. Moreover Joule heating is accounted. Study is done for the spherical and cylindrical nanoparticles. Viscous dissipation and heat generation/absorption are also considered. Convective boundary conditions are utilized. System of equations are solved numerically. The results are analyzed for the various parameters of interest.

## Modeling

**Fig 1:**
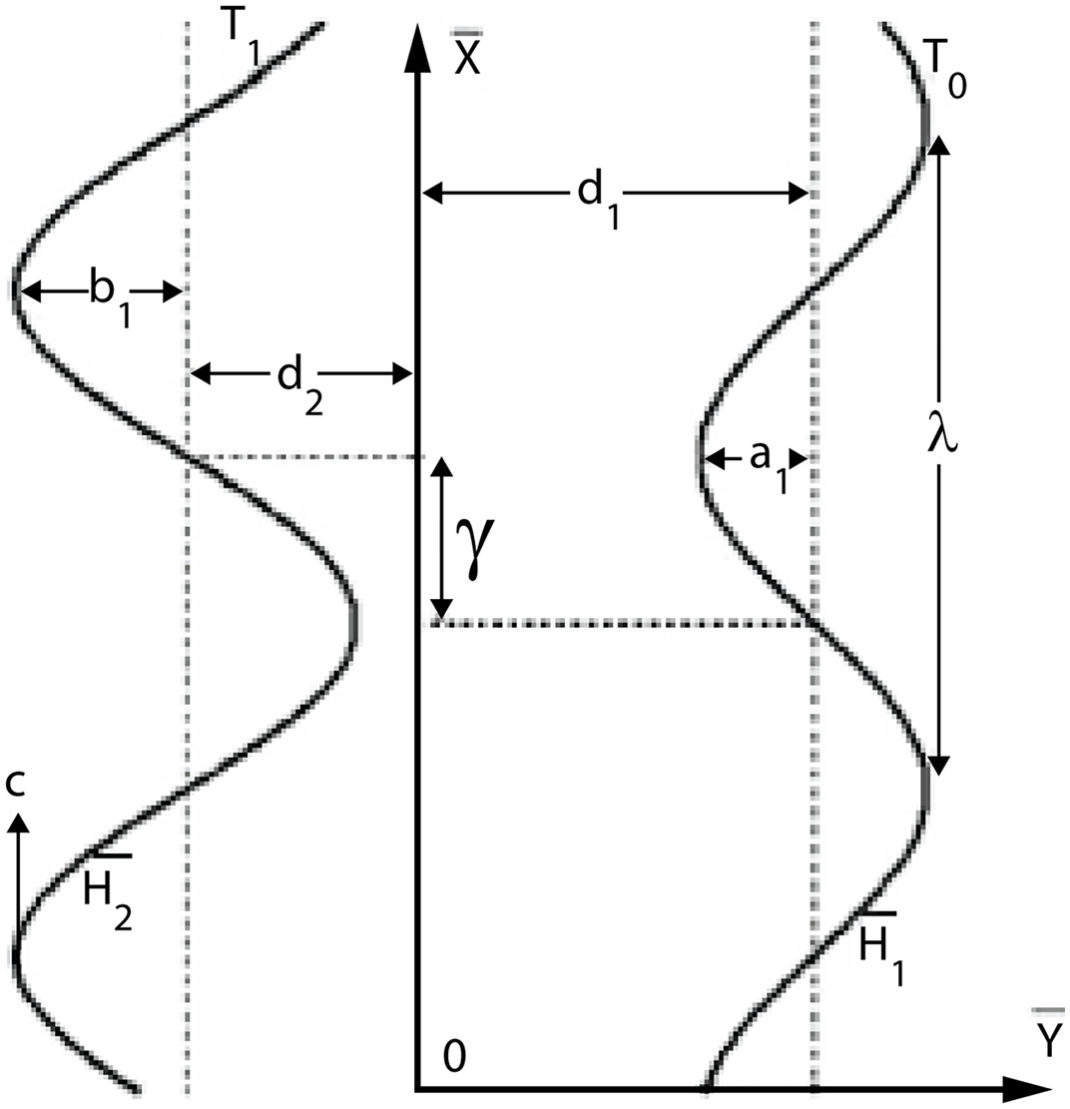
Problem sketch.

An incompressible water-based nanofluid filling an asymmetric channel of width d_1_+d_2_ (see Fig 1) is considered. Nanofluids are the suspension of Titanium oxide or titania (TiO_2_), Aluminum oxide or Alumina (Al_2_O_3_), Copper oxide (CuO), Copper (Cu) and Silver (Ag) and water. Moreover base fluid and nanoparticles are considered thermally consistent with respect to each other. Magnetic field of strength B_0_ is applied in a direction normal to flow. Induced magnetic field for small magnetic Reynolds number is ignored.

The Lorentz force is given by

F = J×B,

where **B** = [0,0,B_0_] and ***J*** denotes the applied magnetic field and current density respectively. By considering the Hall effects the current density can be represented as follows:

$$J\;=\;\sigma_{eff}\left[E+V\times B-\frac{1}{en_{e}}\left[J\times B\right]\right].$$

Here *σ*_*eff*_ denotes the effective electric conductivity of nanofluid, **E** is the electric field, the velocity field $V\;=\;\left[\bar{U}(\bar{X},\bar{Y},\bar{t}\right),\;\bar{V}\left(\bar{X},\bar{Y},\bar{t}\right),0]$, *e* represents the electron charge and *n*_*e*_ the number density of free electrons. Here applied and the induced electric fields are taken zero and thus

$$J\;=\;\sigma_{eff}\left[V\times B-\frac{1}{en_{e}}\left[J\times B\right]\right].$$

The Lorentz force is given by

$$F\;=\;\left[\frac{\sigma_{eff}B_{0}{}^{2}}{1+{\left(\frac{\sigma_{eff}B_{0}}{en_{e}}\right)}^{2}}\left(-\bar{U}+\left(\frac{\sigma_{eff}B_{0}}{en_{e}}\right)\bar{V}\right),\;\frac{-\sigma_{eff}B_{0}{}^{2}}{1+{\left(\frac{\sigma_{eff}B_{0}}{en_{e}}\right)}^{2}}\left(\bar{V}+\left(\frac{\sigma_{eff}B_{0}}{en_{e}}\right)\bar{U}\right),0\right].$$

The effective electric conductivity of nanofluid for two-phase flow model is defined below [32]:

$$\frac{\sigma_{eff}}{\sigma_{f}}=1+\frac{3\left(\frac{\sigma_{p}}{\sigma_{f}}-1\right)\phi }{\left(\frac{\sigma_{p}}{\sigma_{f}}+2\right)-\left(\frac{\sigma_{p}}{\sigma_{f}}-1\right)\phi }.$$

Here *σ*_*p*_ and *σ*_*f*_ are the electric conductivity of nanoparticles and water respectively and *ϕ* denote the nanoparticle volume fraction. Now we have

$$F\;=\;\left[\frac{A_{1}\sigma_{f}B_{0}{}^{2}}{1+{\left(A_{1}m\right)}^{2}}\left(-\bar{U}+A_{1}m\bar{V}\right),\;\frac{-A_{1}\sigma_{f}B_{0}{}^{2}}{1+{\left(A_{1}m\right)}^{2}}\left(\bar{V}+A_{1}m\bar{U}\right),0\right],$$

where *A*_1_ and the Hall parameter *m* is defined by:

$$m=\frac{\sigma_{f}B_{0}}{en_{e}},\;A_{1}=1+\frac{3\left(\frac{\sigma_{p}}{\sigma_{f}}-1\right)\phi }{\left(\frac{\sigma_{p}}{\sigma_{f}}+2\right)-\left(\frac{\sigma_{p}}{\sigma_{f}}-1\right)\phi }.$$

The Joule heating is

$$\frac{1}{\sigma_{eff}}J.J=\frac{A_{1}\sigma_{f}B_{0}{}^{2}}{1+{\left(A_{1}m\right)}^{2}}\left({\bar{U}}^{2}+{\bar{V}}^{2}\right).$$

Now we select the Cartesian coordinate system such that $\bar{X}$ axis is along the length of channel and $\bar{Y}$ axis is perpendicular to it. Sinusoidal wave with constant speed *c* and wavelength λ propagate along the channel walls and hence generates the peristaltic flow. The shapes of travelling waves are represented by following relations:

$$\begin{array}{l} {\bar{H}}_{1}\left(\bar{X},\bar{t}\right)=d_{1}+a_{1}\text{cos}\frac{2\pi }{\lambda }\left(\bar{X}-c\bar{t}\right),\\ {\bar{H}}_{2}\left(\bar{X},\bar{t}\right)=-d_{2}-b_{1}\text{cos}\left(\frac{2\pi }{\lambda }\left(\bar{X}-c\bar{t}\right)+\gamma \right), \end{array}$$ (1)

where *a*_1_ and *b*_1_ are the wave amplitudes, *γ* the phase difference in the range 0 ≤ *γ* ≤ *π*.

For the considered flow analysis the continuity equation is

$$\frac{\partial \bar{U}}{\partial \bar{X}}+\frac{\partial \bar{V}}{\partial \bar{Y}}=\;0.$$ (2)

Velocity components in $\bar{X}$ and $\bar{Y}$ directions are

$$\rho_{eff}\left(\frac{\partial }{\partial \bar{t}}+\bar{U}\frac{\partial }{\partial \bar{X}}+\bar{V}\frac{\partial }{\partial \bar{Y}}\right)\bar{U}=-\frac{\partial \bar{P}}{\partial \bar{X}}+\mu_{eff}\left[\frac{\partial^{2}\bar{U}}{\partial {\bar{X}}^{2}}+\frac{\partial^{2}\bar{U}}{\partial {\bar{Y}}^{2}}\right]-\frac{A_{1}\sigma_{f}B_{0}{}^{2}}{1+{\left(A_{1}m\right)}^{2}}\left(\bar{U}-A_{1}m\bar{V}\right)+\;\;g{\left(\rho \beta \right)}_{eff}\left(T-T_{m}\right),$$ (3)

$$\rho_{eff}\left(\frac{\partial }{\partial \bar{t}}+\bar{U}\frac{\partial }{\partial \bar{X}}+\bar{V}\frac{\partial }{\partial \bar{Y}}\right)\bar{V}=-\frac{\partial \bar{P}}{\partial \bar{Y}}+\mu_{eff}\left[\frac{\partial^{2}\bar{V}}{\partial {\bar{X}}^{2}}+\frac{\partial^{2}\bar{V}}{\partial {\bar{Y}}^{2}}\right]-\frac{A_{1}\sigma_{f}B_{0}{}^{2}}{1+{\left(A_{1}m\right)}^{2}}\left(\bar{V}+A_{1}m\bar{U}\right).$$ (4)

The energy equation with viscous dissipation and Joule heating effects is

$${\left(\rho C\right)}_{eff}\left(\frac{\partial }{\partial \bar{t}}+\bar{U}\frac{\partial }{\partial \bar{X}}+\bar{V}\frac{\partial }{\partial \bar{Y}}\right)T=\;K_{eff}\left[\frac{\partial^{2}T}{\partial {\bar{X}}^{2}}+\frac{\partial^{2}T}{\partial {\bar{Y}}^{2}}\right]\;+\;\Phi \;+\;\mu_{eff}\left[2\left({\left(\frac{\partial \bar{U}}{\partial \bar{X}}\right)}^{2}+{\left(\frac{\partial \bar{V}}{\partial \bar{Y}}\right)}^{2}\right)+{\left(\frac{\partial \bar{U}}{\partial \bar{Y}}+\frac{\partial \bar{V}}{\partial \bar{X}}\right)}^{2}\right]\;+\;\frac{A_{1}\sigma_{f}B_{0}{}^{2}}{1+{\left(A_{1}m\right)}^{2}}\left({\bar{U}}^{2}+{\bar{V}}^{2}\right).$$ (5)

Here $\bar{P}\left(\bar{X},\bar{Y},\bar{t}\right)$ denotes the pressure, *g* denotes the acceleration due to gravity, $T_{m}\;=\;\frac{T_{0}+T_{1}}{2}$ the mean temperature and Φ is the dimensional heat generation/absorption parameter. The effective density *ρ*_*eff*_, specific heat *C*_*eff*_, effective viscosity and thermal expansion coefficient for the two-phase flow model are [33]:

$$\rho_{eff}=\left(1-\phi \right)\rho_{f}+\phi \rho_{p},\;{\left(\rho C\right)}_{eff}=\left(1-\phi \right){\left(\rho C\right)}_{f}+\phi {\left(\rho C\right)}_{p},$$

$${\left(\rho \beta \right)}_{eff}=\left(1-\phi \right)\rho_{f}\beta_{f}+\phi \rho_{p}\beta_{p},\;\mu_{eff}=\frac{\mu_{f}}{{\left(1-\phi \right)}^{2.5}}.$$

The effective thermal conductivity for Maxwell's and Hamilton-Crosser's model is defined as follows:

$$\frac{K_{eff}}{K_{f}}=\frac{K_{p}+2K_{f}-2\phi \left(K_{f}-K_{p}\right)}{K_{p}+2K_{f}+\phi \left(K_{f}-K_{p}\right)},$$ (6)

[11] for Maxwell’s model and

$$\frac{K_{eff}}{K_{f}}=\frac{K_{p}+\left(n-1\right)K_{f}-\left(n-1\right)\phi \left(K_{f}-K_{p}\right)}{K_{p}+\left(n-1\right)K_{f}+\phi \left(K_{f}-K_{p}\right)},$$ (7)

[12] for Hamilton-Crosser’s model

where the subscripts *p* and *f* indicate the nanoparticle and fluid phases respectively.

Here *n* in Hamilton Crosser's represents the shape factor and its value is n = 3/*ψ* where *ψ* represents the sphericity of the nanoparticles. Here *ψ* = 0.5 and 1 correspond to the cylindrical and spherical nanoparticles. For *n* = 3 the Hamilton-Crosser's model reduces to the Maxwell's model.

The transformation between fixed and wave frames are given as follows:

$$\begin{array}{l} \bar{x}=\bar{X}-c\bar{t},\;\bar{y}=\bar{Y,}\;\bar{u}\left(\bar{x},\bar{y}\right)=\bar{U}\left(\bar{X},\bar{Y},\bar{t}\right)-c,\\ \bar{\;v}\left(\bar{x},\bar{y}\right)=\bar{V}\left(\bar{X},\bar{Y},\bar{t}\right),\;\;\bar{p}\left(\bar{x},\bar{y}\right)=\bar{P}\left(\bar{X},\bar{Y},\bar{t}\right), \end{array}$$ (8)

After using the above transformations, the Eqs 2–5 become:

$$\frac{\partial \bar{u}}{\partial \bar{x}}+\frac{\partial \bar{v}}{\partial \bar{y}}\;=\;0,$$ (9)

$$\left(\left(1-\phi \right)\rho_{f}+\phi \rho_{p}\right)\left(\left(\bar{u}+c\right)\frac{\partial }{\partial \bar{x}}+\bar{v}\frac{\partial }{\partial \bar{y}}\right)\left(\bar{u}+c\right)=-\frac{\partial \bar{p}}{\partial \bar{x}}+\frac{\mu_{f}}{{\left(1-\phi \right)}^{2.5}}\left[\frac{\partial^{2}\bar{u}}{\partial {\bar{x}}^{2}}+\frac{\partial^{2}\bar{u}}{\partial {\bar{y}}^{2}}\right]-\frac{A_{1}\sigma_{f}B_{0}{}^{2}}{1+{\left(A_{1}m\right)}^{2}}\left(\left(\bar{u}+c\right)-A_{1}m\bar{v}\right)+g\left(\left(1-\phi \right)\rho_{f}\beta_{f}+\phi \rho_{p}\beta_{p}\right)\left(T-T_{m}\right),$$ (10)

$$\left(\left(1-\phi \right)\rho_{f}+\phi \rho_{p}\right)\left(\left(\bar{u}+c\right)\frac{\partial }{\partial \bar{x}}+\bar{v}\frac{\partial }{\partial \bar{y}}\right)\left(\bar{v}\right)=-\frac{\partial \bar{p}}{\partial \bar{y}}+\frac{\mu_{f}}{{\left(1-\phi \right)}^{2.5}}\left[\frac{\partial^{2}\bar{v}}{\partial {\bar{x}}^{2}}+\frac{\partial^{2}\bar{v}}{\partial {\bar{y}}^{2}}\right]-\frac{A_{1}\sigma_{f}B_{0}{}^{2}}{1+{\left(A_{1}m\right)}^{2}}\left(\bar{v}+A_{1}m\left(\bar{u}+c\right)\right),$$ (11)

$$\left(\left(1-\phi \right){\left(\rho C\right)}_{f}+\phi {\left(\rho C\right)}_{p}\right)\left(\left(\bar{u}+c\right)\frac{\partial }{\partial \bar{x}}+\bar{v}\frac{\partial }{\partial \bar{y}}\right)T=K_{eff}\left[\frac{\partial^{2}T}{\partial {\bar{x}}^{2}}+\frac{\partial^{2}T}{\partial {\bar{y}}^{2}}\right]+\Phi +\frac{\mu_{f}}{{\left(1-\phi \right)}^{2.5}}\left[2\left({\left(\frac{\partial \bar{u}}{\partial \bar{x}}\right)}^{2}+{\left(\frac{\partial \bar{v}}{\partial \bar{y}}\right)}^{2}\right)+{\left(\frac{\partial \bar{u}}{\partial \bar{y}}+\frac{\partial \bar{v}}{\partial \bar{x}}\right)}^{2}\right]+\frac{A_{1}\sigma_{f}B_{0}{}^{2}}{1+{\left(A_{1}m\right)}^{2}}\left({\left(\bar{u}+c\right)}^{2}+{\bar{v}}^{2}\right).$$ (12)

Numerical values of the thermophysical parameters of water and nanoparticles are mentioned in Table 1.

**Table 1:** Thermophysical parameters of water and nanoparticles [34].

	ρ (kg m^-3^)	C_p_ (j kg^-1^ K^-1^)	K (Wm^-1^K^-1)^	β (l/k) × 10^−6^	σ (Ω.*m*)^-1^
H_2_ O	997.1	4179	0.613	210	0.05
TiO_2_	4250	686.2	8.9538	9.0	1 × 10^−12^
Al_2_ O_3_	3970	765	40	8.5	1 × 10^−10^
CuO	6320	531.8	76.5	18.0	2.7 × 10^−8^
Cu	8933	385	401	16.7	5.96 × 10^7^
Ag	10500	235	429	18.9	6.3 × 10^7^

Non-dimensional quantities used in the above Eqs are

$$\begin{array}{l} x=\frac{\bar{x}}{\lambda },\;\;y=\frac{\bar{y}}{d_{1}},\;\;u=\frac{\bar{u}}{c},\;\;v=\frac{\bar{v}}{c\delta },\;\;\delta =\frac{d_{1}}{\lambda },\;\;h_{1}=\frac{\bar{H_{1}}}{d_{1}},\;\;h_{2}=\frac{\bar{H_{2}}}{d_{1}},\\ d=\frac{d_{2}}{d_{1}},\;a=\frac{a_{1}}{d_{1}},\;b=\frac{b_{1}}{d_{1}},\;p=\frac{d_{1}{}^{2}\bar{p}}{c\lambda \mu_{f}},\;\theta =\frac{T-T_{m}}{T_{1-}T_{0}},\;Re=\frac{\rho_{f}cd_{1}}{\mu_{f}},\\ Pr=\frac{\mu_{f}C_{f}}{K_{f}},\;Ec=\frac{c^{2}}{C_{f}\left(T_{1-}T_{0}\right)},\;Br=PrEc,\;Gr=\frac{g\rho_{f}\beta_{f}\left(T_{1-}T_{0}\right)d_{1}{}^{2}}{c\mu_{f}},\\ M=\sqrt{\frac{\sigma_{f}}{\mu_{f}}}B_{0}d_{1},\;\varepsilon =\frac{d_{1}{}^{2}\Phi }{\left(T_{1-}T_{0}\right)K_{f}},\;u=\frac{\partial \psi }{\partial y},\;v=-\frac{\partial \psi }{\partial x}, \end{array}$$ (13)

in which Re, Pr, Ec, Br, Gr, M represent the Reynolds, Prandtl, Eckert, Brinkman, Grashoff and Hartman numbers respectively whereas ε denotes the dimensionless heat generation/absorption.

After utilizing the lubrication approach, Eqs 10–12 take the form

$$\frac{\partial p}{\partial x}=\frac{1}{{\left(1-\phi \right)}^{2.5}}\frac{\partial^{3}\psi }{\partial y^{3}}+A_{2}Gr\theta -\frac{A_{1}M^{2}}{1+{\left(A_{1}m\right)}^{2}}\left(\frac{\partial \psi }{\partial y}+1\right),$$ (14)

$$\frac{\partial p}{\partial y}=0,$$ (15)

$$A_{3}\frac{\partial^{2}\theta }{\partial y^{2}}+\frac{Br}{{\left(1-\phi \right)}^{2.5}}{\left(\frac{\partial^{2}\psi }{\partial y^{2}}\right)}^{2}+\frac{BrA_{1}M^{2}}{1+{\left(A_{1}m\right)}^{2}}{\left(\frac{\partial \psi }{\partial y}+1\right)}^{2}+\varepsilon =0,$$ (16)

in which

$$A_{1}\;=\;1+\frac{3\left(\frac{\sigma_{p}}{\sigma_{f}}-1\right)\phi }{\left(\frac{\sigma_{p}}{\sigma_{f}}+2\right)-\left(\frac{\sigma_{p}}{\sigma_{f}}-1\right)\phi },\;A_{2}\;=\;1-\phi +\phi \left(\frac{{\left(\rho \beta \right)}_{p}}{{\left(\rho \beta \right)}_{f}}\right),$$

$$A_{3}\;=\;\frac{K_{p}+2K_{f}-2\phi \left(K_{f}-K_{p}\right)}{K_{p}+2K_{f}+\phi \left(K_{f}-K_{p}\right)},$$

[11] for Maxwell’s model and

$$A_{3}=\frac{K_{p}+\left(n-1\right)K_{f}-\left(n-1\right)\phi \left(K_{f}-K_{p}\right)}{K_{p}+\left(n-1\right)K_{f}+\phi \left(K_{f}-K_{p}\right)}$$ (17)

[12] for Hamilton-Crosser’s model.

Dimensionless flow rates in the laboratory $\eta \;\left(=\;\frac{\bar{Q}}{cd_{1}}\right)$ and wave frames $F\;\left(=\;\frac{\bar{q}}{cd_{1}}\right)$ are related by

η=F+1+d, (18)

in which $\bar{Q}$ and $\bar{q}$ denote the dimensional flow rates in the laboratory and wave frames respectively and

$$F=\int_{h2}^{h1}\frac{\partial \psi }{\partial y}dy.$$ (19)

The no slip and convective conditions on the boundary are represented in the forms

$$\begin{array}{l} \bar{U}=0,\;\;K_{eff}\frac{\partial T}{\partial y}=-B_{1}\left(T-T_{0}\right)\;\;at\;\bar{Y}={\bar{H}}_{1}\left(\bar{X},\bar{t}\right),\\ \bar{U}=0,\;\;K_{eff}\frac{\partial T}{\partial y}=-B_{2}\left(T_{1}-T\right)\;\;at\;\bar{Y}={\bar{H}}_{2}\left(\bar{X},\bar{t}\right), \end{array}$$ (20)

where, *B*_1_ and *B*_2_ are heat transfer coefficients at the walls respectively.

The boundary conditions in dimensionless form are defined by:

$$\begin{array}{l} \psi =\frac{F}{2},\;\;\;\frac{\partial \psi }{\partial y}=-1,\;\;\;\frac{\partial \theta }{\partial y}+\frac{Bi_{1}}{\left(\frac{K_{eff}}{K_{f}}\right)}\left(\theta +\frac{1}{2}\right)=0\;\;at\;y=h_{1},\\ \psi =-\frac{F}{2},\;\;\;\frac{\partial \psi }{\partial y}=-1,\;\;\;\frac{\partial \theta }{\partial y}-\frac{Bi_{2}}{\left(\frac{K_{eff}}{K_{f}}\right)}\left(\theta -\frac{1}{2}\right)=0\;\;at\;y=h_{2}. \end{array}$$ (21)

Dimensionless form of the walls shape is defined as follows:

$$\begin{array}{l} h_{1}\left(x\right)=1+a\text{cos}\left(2\pi x\right),\\ h_{2}\left(x\right)=-d-b\text{cos}\left(2\pi x+\gamma \right). \end{array}$$ (22)

Now the Eqs 14–16 and the boundary conditions Eq (21) are solved by using the NDSolve in MATHEMATICA. The obtained results are analyzed in the next section via graphs and bar charts.

## Results and Discussion

In this section we investigate the behavior of different water-based nanofluids for different embedded parameters by considering the two thermal conductivity models. Analysis of velocity, temperature and heat transfer rate at the wall is given due attention.

**Fig 2:**
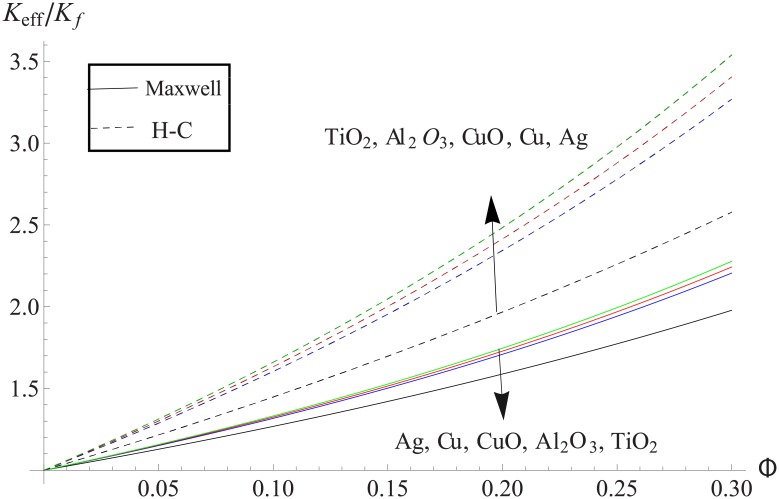
The effective thermal conductivity for Maxwell’s and H-C’s models.

Fig 2 elucidates the effect of volume fraction on the effective thermal conductivity of the various nanofluids consisting of nanoparticles. Higher the effective thermal conductivity is observed for Hamilton-Crosser's model when compared with the Maxwell's model. It is also noticed that the difference between the values get enhanced when we increase the volume fraction ϕ. Moreover for metallic particles such difference is greater than the metallic oxides particles.

**Fig 3:**
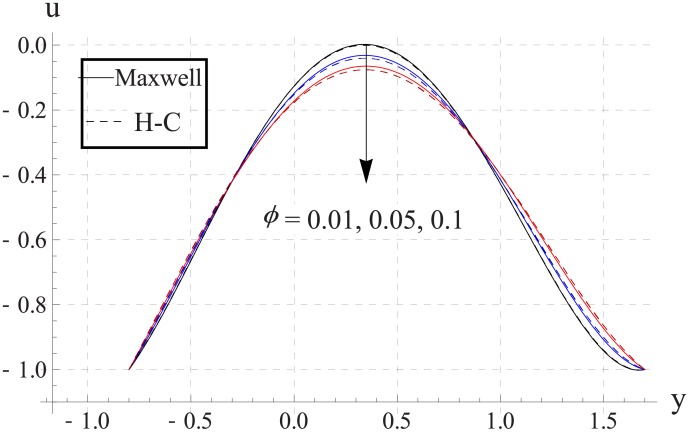
Effect of ϕ on axial velocity for TiO_2_ when a = 0.7, b = 0.6, γ = π/2, d = 0.8, x = 0, η = 0.7, Br = 0.3, m = 2.0, M = 1.0, Bi_1_ = 8, Bi_2_ = 10, Gr = 3.0, ε = 2.5.

**Fig 4:**
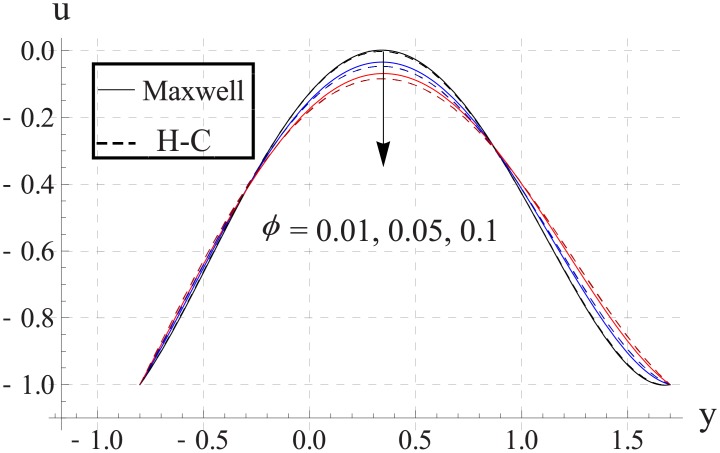
Effect of ϕ on axial velocity for Al_2_O_3_ when a = 0.7, b = 0.6, γ = π/2, d = 0.8, x = 0, η = 0.7, Br = 0.3, m = 2.0, M = 1.0, Bi_1_ = 8, Bi_2_ = 10, Gr = 3.0, ε = 2.5.

**Fig 5:**
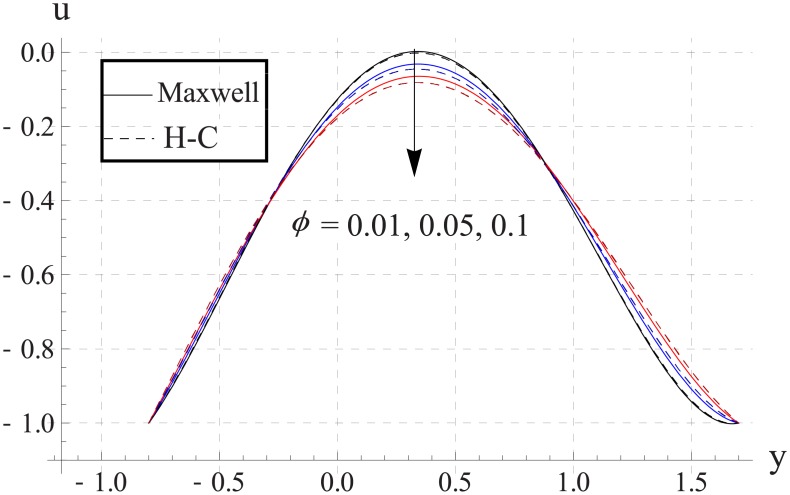
Effect of ϕ on axial velocity for CuO when a = 0.7, b = 0.6, γ = π/2, d = 0.8, x = 0, η = 0.7, Br = 0.3, m = 2.0, M = 1.0, Bi_1_ = 8, Bi_2_ = 10, Gr = 3.0, ε = 2.5.

**Fig 6:**
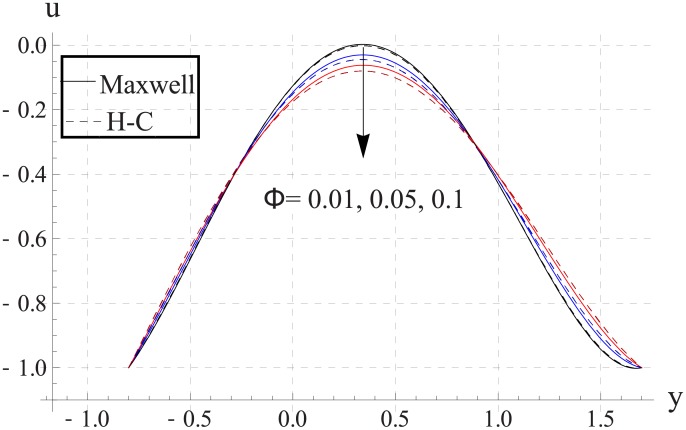
Effect of ϕ on axial velocity for Cu when a = 0.7, b = 0.6, γ = π/2, d = 0.8, x = 0, η = 0.7, Br = 0.3, m = 2.0, M = 1.0, Bi_1_ = 8, Bi_2_ = 10, Gr = 3.0, ε = 2.5.

**Fig 7:**
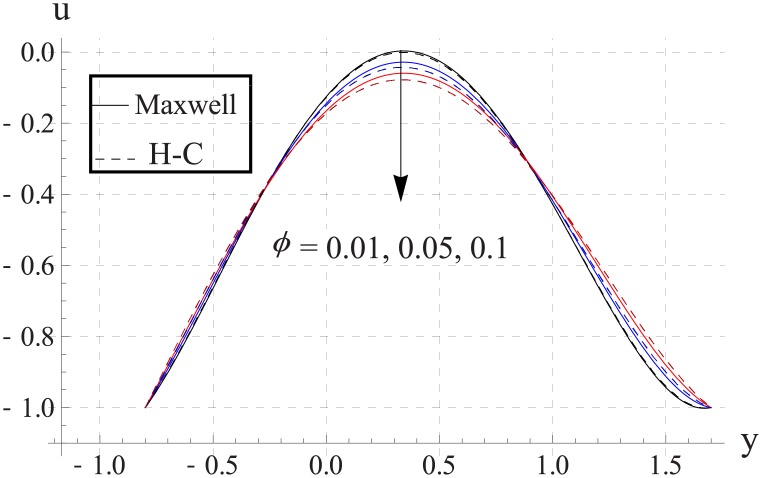
Effect of ϕ on axial velocity for Ag when a = 0.7, b = 0.6, γ = π/2, d = 0.8, x = 0, η = 0.7, Br = 0.3, m = 2.0, M = 1.0, Bi_1_ = 8, Bi_2_ = 10, Gr = 3.0, ε = 2.5.

Figs 3–7 are prepared to study the impact of volume fraction on velocity for five different nanofluids. We can see that with an increase in volume fraction the velocity of the nanofluid near the center of channel decreases. Maximum velocity is higher for Maxwell's model than H-C's model. Such decrease in maximum value of the velocity is due to an increase in the resistance produced by increasing the nanoparticle volume fraction ϕ. Infact an increase in nanoparticle volume fraction greatly enhances the viscosity of the nanofluid. This ultimate reduces the maximum velocity. Moreover with an increase in nanoparticle volume fraction the difference between the values of two models increases. Such difference becomes large when thermal conductivity of the nanoparticles is increased.

**Fig 8:**
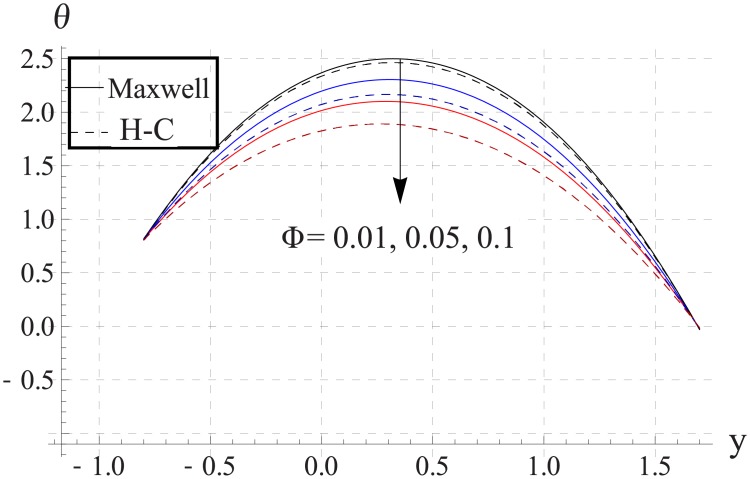
Effect of ϕ on temperature for TiO_2_ when a = 0.7, b = 0.6, γ = π/2, d = 0.8, x = 0, η = 0.7, Br = 0.3, m = 2.0, M = 1.0, Bi_1_ = 8, Bi_2_ = 10, Gr = 3.0, ε = 2.5.

**Fig 9:**
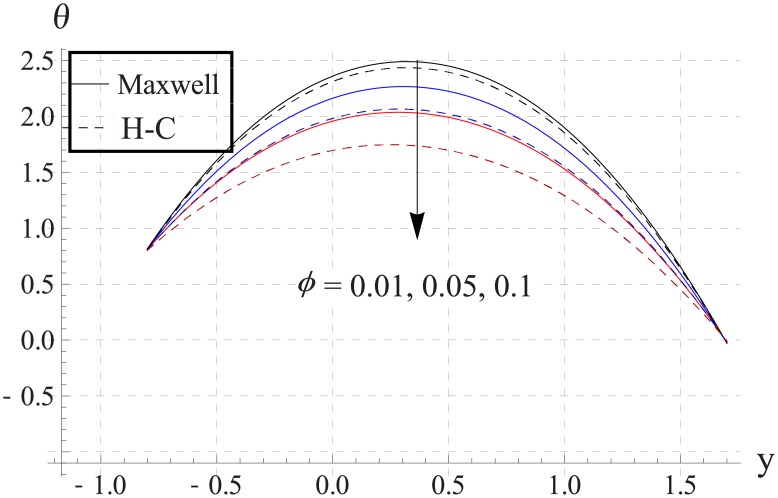
Effect of ϕ on temperature for Al_2_O_3_ when a = 0.7, b = 0.6, γ = π/2, d = 0.8, x = 0, η = 0.7, Br = 0.3, m = 2.0, M = 1.0, Bi_1_ = 8, Bi_2_ = 10, Gr = 3.0, ε = 2.5.

**Fig 10:**
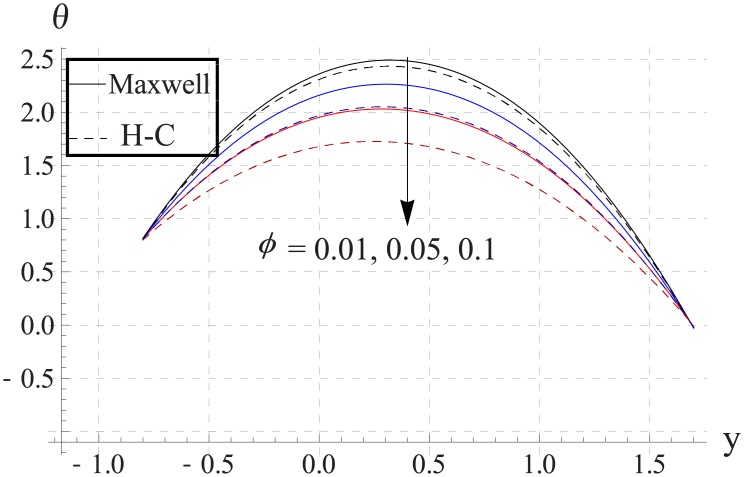
Effect of ϕ on temperature for CuO when a = 0.7, b = 0.6, γ = π/2, d = 0.8, x = 0, η = 0.7, Br = 0.3, m = 2.0, M = 1.0, Bi_1_ = 8, Bi_2_ = 10, Gr = 3.0, ε = 2.5.

**Fig 11:**
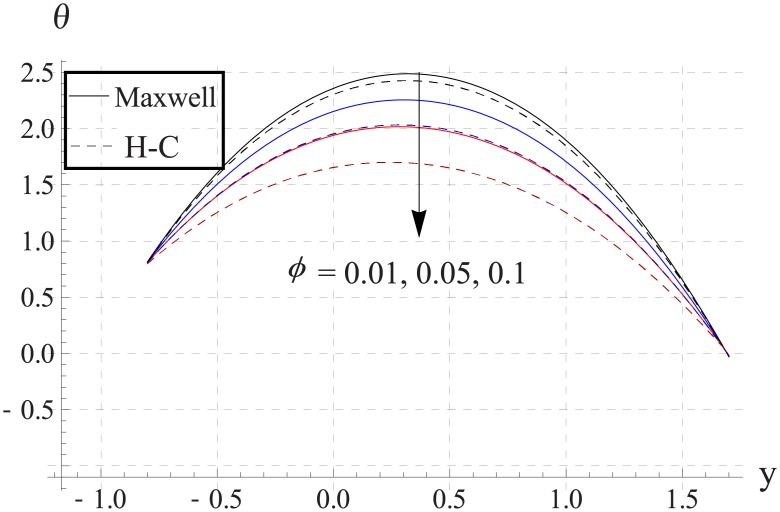
Effect of ϕ on temperature for Cu when a = 0.7, b = 0.6, γ = π/2, d = 0.8, x = 0, η = 0.7, Br = 0.3, m = 2.0, M = 1.0, Bi_1_ = 8, Bi_2_ = 10, Gr = 3.0, ε = 2.5.

**Fig 12:**
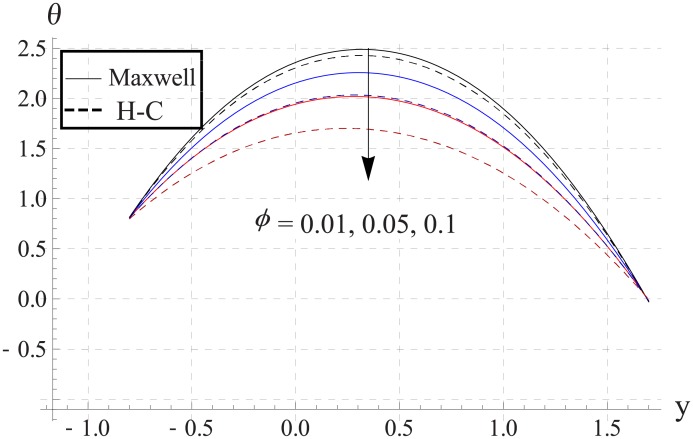
Effect of ϕ on temperature for Ag when a = 0.7, b = 0.6, γ = π/2, d = 0.8, x = 0, η = 0.7, Br = 0.3, m = 2.0, M = 1.0, Bi_1_ = 8, Bi_2_ = 10, Gr = 3.0, ε = 2.5.

In order to analyze the impact of volume fraction on temperature, Figs 8–12 are sketched. Increase in volume fraction of nanofluid causes reduction in temperature. The values computed by using the Maxwell's thermal conductivity is greater than the H-C's model. The reason behind such behavior is the fact that an increase in the effective thermal conductivity causes the increase in heat transfer rate across the boundaries. For the cylindrical nanoparticles this heat transfer rate increases more so values of temperature for H-C's model is less than the Maxwell's model. Also the difference between the values of two models is larger when we increase the nanoparticle volume fraction. Moreover for the particles with high values of thermal conductivities this gap between the values of two models increases.

**Fig 13:**
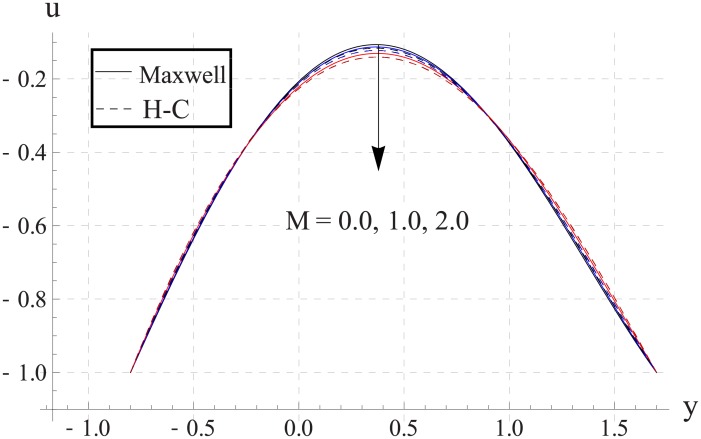
Effect of M on axial velocity for TiO_2_ when a = 0.7, b = 0.6, γ = π/2, d = 0.8, x = 0, η = 0.7, Br = 0.3, m = 0.8, ϕ = 0.2, Bi_1_ = 8, Bi_2_ = 10, Gr = 3.0, ε = 2.5.

**Fig 14:**
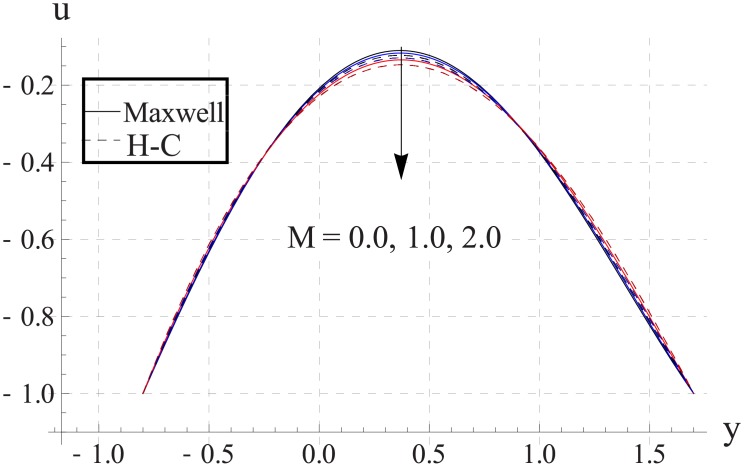
Effect of M on axial velocity for Al_2_O_3_ when a = 0.7, b = 0.6, γ = π/2, d = 0.8, x = 0, η = 0.7, Br = 0.3, m = 0.8, ϕ = 0.2, Bi_1_ = 8, Bi_2_ = 10, Gr = 3.0, ε = 2.5.

**Fig 15:**
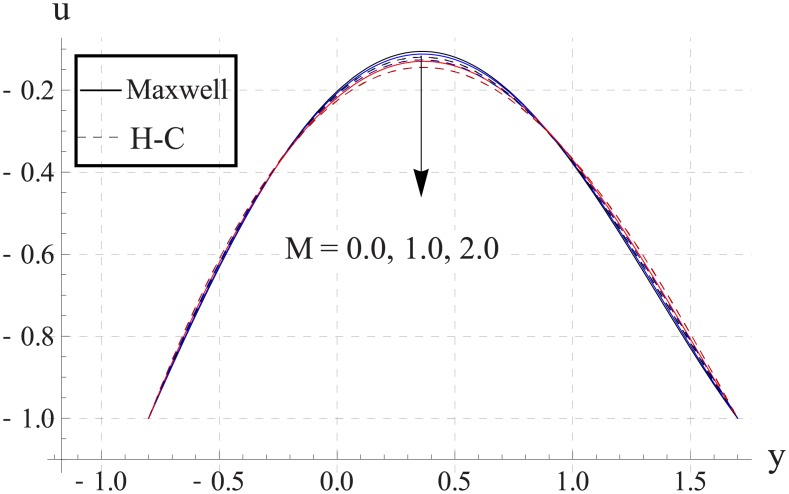
Effect of M on axial velocity for CuO when a = 0.7, b = 0.6, γ = π/2, d = 0.8, x = 0, η = 0.7, Br = 0.3, m = 0.8, ϕ = 0.2, Bi_1_ = 8, Bi_2_ = 10, Gr = 3.0, ε = 2.5.

**Fig 16:**
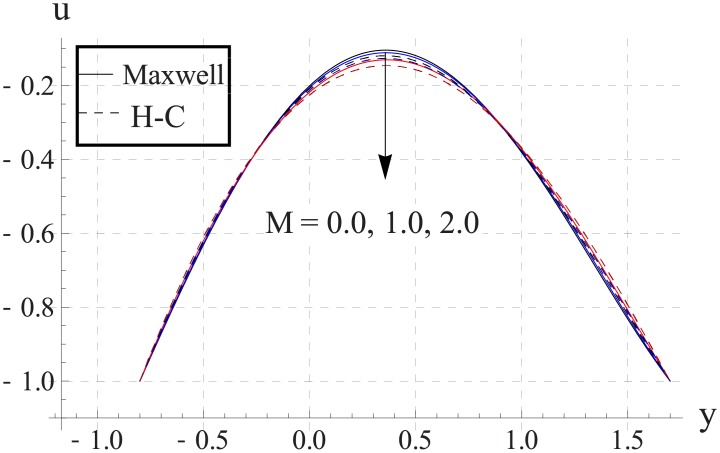
Effect of M on axial velocity for Cu when a = 0.7, b = 0.6, γ = π/2, d = 0.8, x = 0, η = 0.7, Br = 0.3, m = 0.8, ϕ = 0.2, Bi_1_ = 8, Bi_2_ = 10, Gr = 3.0, ε = 2.5.

**Fig 17:**
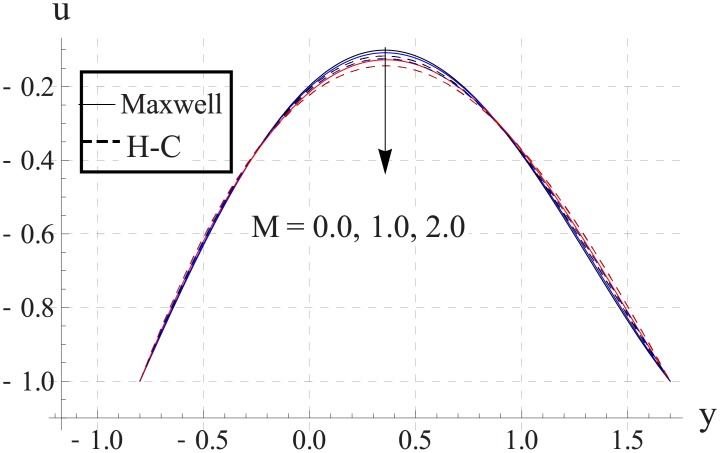
Effect of M on axial velocity for Ag when a = 0.7, b = 0.6, γ = π/2, d = 0.8, x = 0, η = 0.7, Br = 0.3, m = 0.8, ϕ = 0.2, Bi_1_ = 8, Bi_2_ = 10, Gr = 3.0, ε = 2.5.

Figs 13–17 are sketched for the impact of Hartman number on the axial velocity. With increase in Hartman number or increase in the applied magnetic field the maximum velocity of the nanofluid decreases. The resistive nature of magnetic force is responsible for such behavior. The difference between the values of the two models is enhanced when the values of magnetic field strength and thermal conductivity are increased.

**Fig 18:**
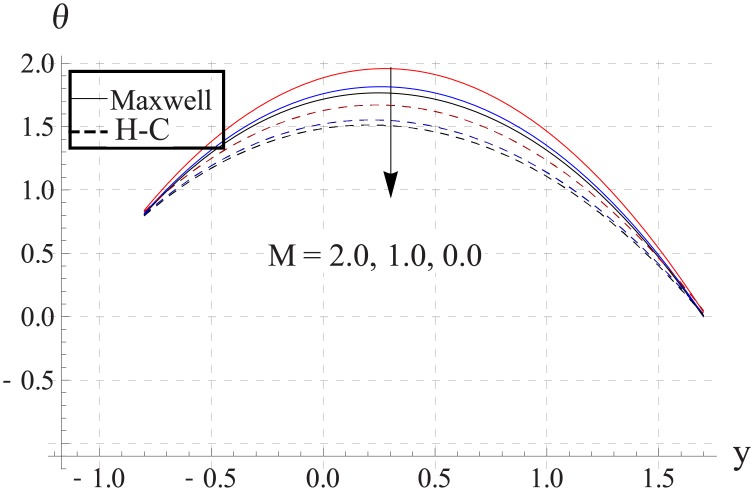
Effect of M on temperature for TiO_2_ when a = 0.7, b = 0.6, γ = π/2, d = 0.8, x = 0, η = 0.7, Br = 0.3, m = 0.8, ϕ = 0.2, Bi_1_ = 8, Bi_2_ = 10, Gr = 3.0, ε = 2.5.

**Fig 19:**
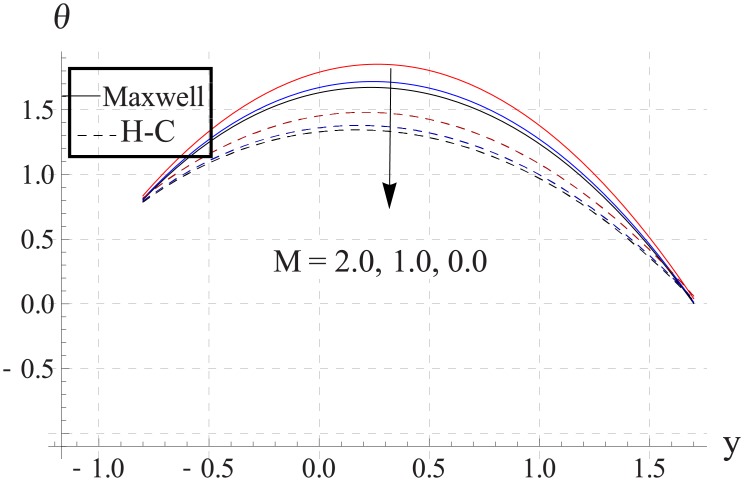
Effect of M on temperature for Al_2_O_3_ when a = 0.7, b = 0.6, γ = π/2, d = 0.8, x = 0, η = 0.7, Br = 0.3, m = 0.8, ϕ = 0.2, Bi_1_ = 8, Bi_2_ = 10, Gr = 3.0, ε = 2.5.

**Fig 20:**
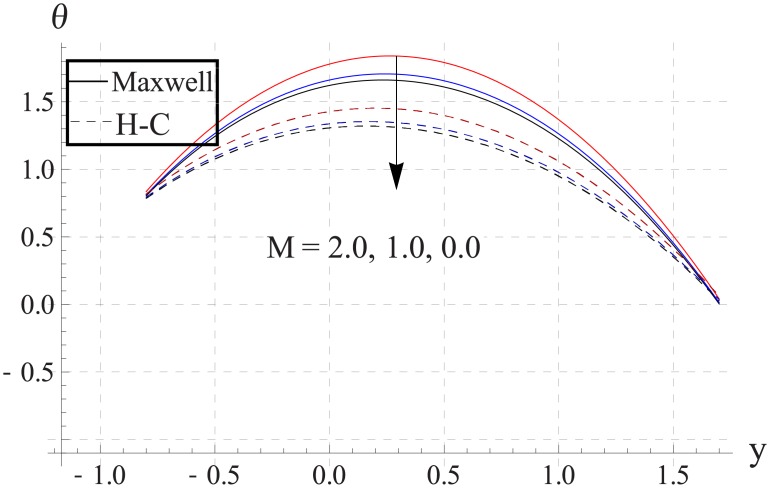
Effect of M on temperature for CuO when a = 0.7, b = 0.6, γ = π/2, d = 0.8, x = 0, η = 0.7, Br = 0.3, m = 0.8, ϕ = 0.2, Bi_1_ = 8, Bi_2_ = 10, Gr = 3.0, ε = 2.5.

**Fig 21:**
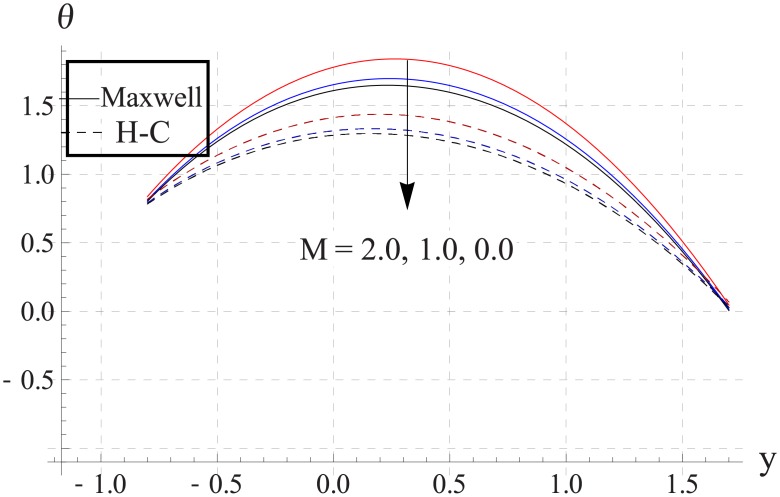
Effect of M on temperature for Cu when a = 0.7, b = 0.6, γ = π/2, d = 0.8, x = 0, η = 0.7, Br = 0.3, m = 0.8, ϕ = 0.2, Bi_1_ = 8, Bi_2_ = 10, Gr = 3.0, ε = 2.5.

**Fig 22:**
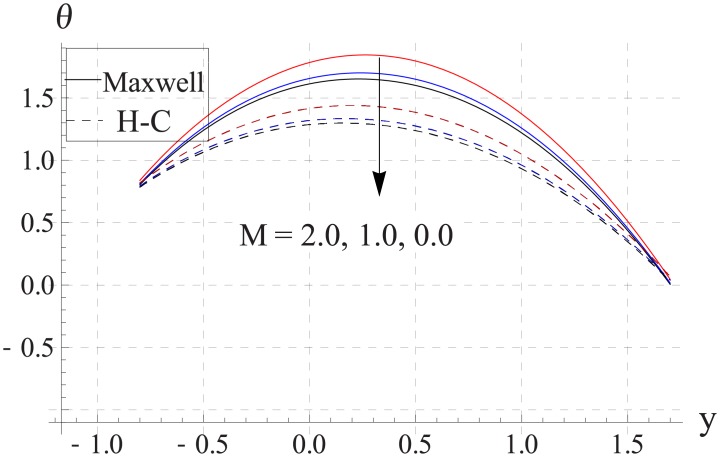
Effect of M on temperature for Ag when a = 0.7, b = 0.6, γ = π/2, d = 0.8, x = 0, η = 0.7, Br = 0.3, m = 0.8, ϕ = 0.2, Bi_1_ = 8, Bi_2_ = 10, Gr = 3.0, ε = 2.5.

Figs 18–22 study the impact of Hartman number on the temperature. We observed that with an increase in the magnetic field strength the temperature of the nanofluid increases. Such increase in the temperature results from Ohmic heating (Joule heating). Also difference between two models increases with an increase in applied magnetic field. Such difference is greater for the metallic nanoparticles than the metallic oxides ones.

**Fig 23:**
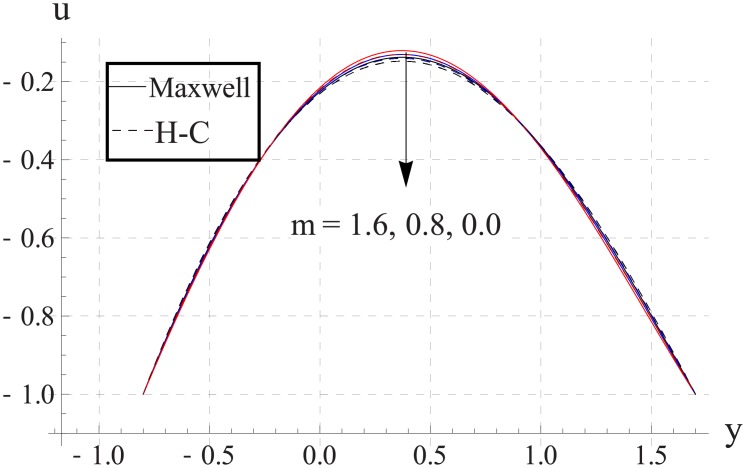
Effect of m on axial velocity for TiO_2_ when a = 0.7, b = 0.6, γ = π/2, d = 0.8, x = 0, η = 0.7, Br = 0.3, ϕ = 0.2, M = 2.0, Bi_1_ = 8, Bi_2_ = 10, Gr = 3.0, ε = 2.5.

**Fig 24:**
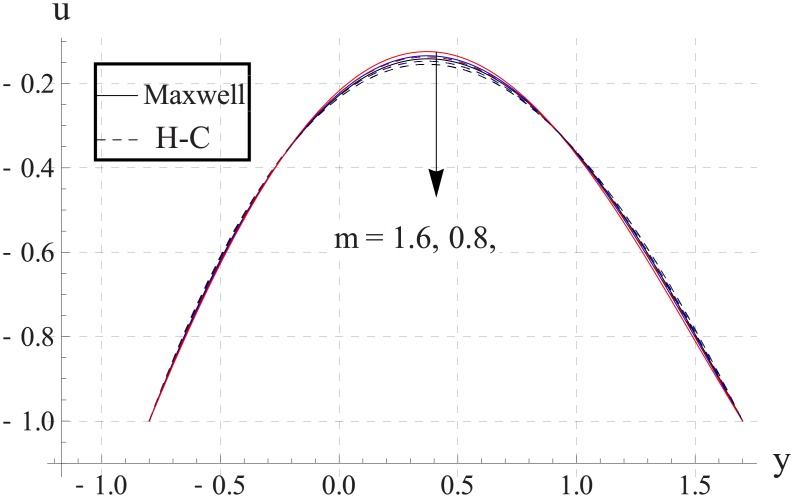
Effect of m on axial velocity for Al_2_O_3_ when a = 0.7, b = 0.6, γ = π/2, d = 0.8, x = 0, η = 0.7, Br = 0.3, ϕ = 0.2, M = 2.0, Bi_1_ = 8, Bi_2_ = 10, Gr = 3.0, ε = 2.5.

**Fig 25:**
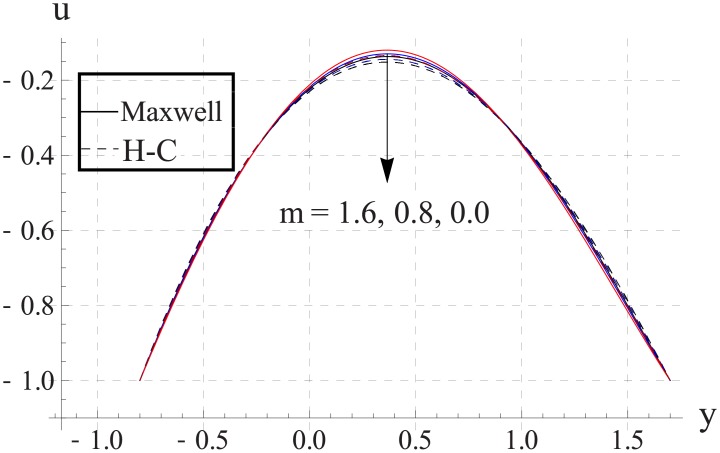
Effect of m on axial velocity for CuO when a = 0.7, b = 0.6, γ = π/2, d = 0.8, x = 0, η = 0.7, Br = 0.3, ϕ = 0.2, M = 2.0, Bi_1_ = 8, Bi_2_ = 10, Gr = 3.0, ε = 2.5.

**Fig 26:**
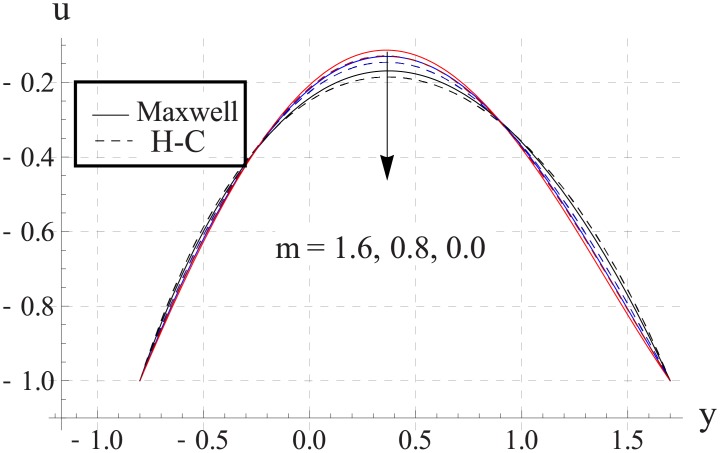
Effect of m on axial velocity for Cu when a = 0.7, b = 0.6, γ = π/2, d = 0.8, x = 0, η = 0.7, Br = 0.3, ϕ = 0.2, M = 2.0, Bi_1_ = 8, Bi_2_ = 10, Gr = 3.0, ε = 2.5.

**Fig 27:**
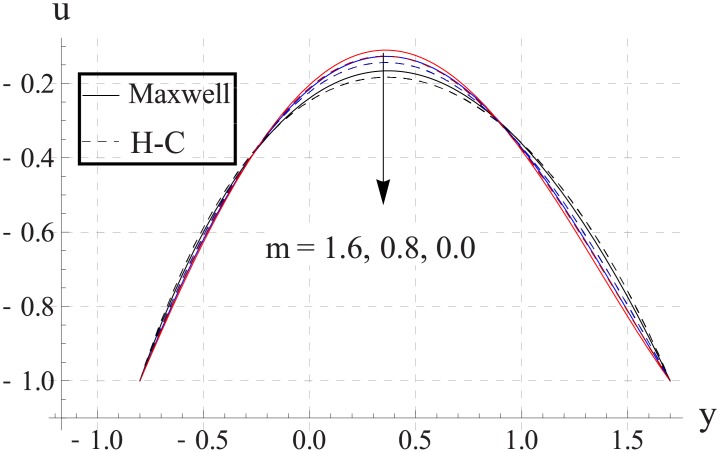
Effect of m on axial velocity for Ag when a = 0.7, b = 0.6, γ = π/2, d = 0.8, x = 0, η = 0.7, Br = 0.3, ϕ = 0.2, M = 2.0, Bi_1_ = 8, Bi_2_ = 10, Gr = 3.0, ε = 2.5.

Figs 23–27 show the behavior of Hall parameter on velocity. We noticed that maximum velocity of nanofluid increases for larger Hall parameter. This indicates that the Hall parameter resists the change in the fluid caused by an increase in the strength of applied magnetic field. In all of the above mentioned quantities the value of Maxwell's model is greater than the H-C's model. Furthermore the difference between the two models is larger when we increase the thermal conductivity of the nanoparticles.

**Fig 28:**
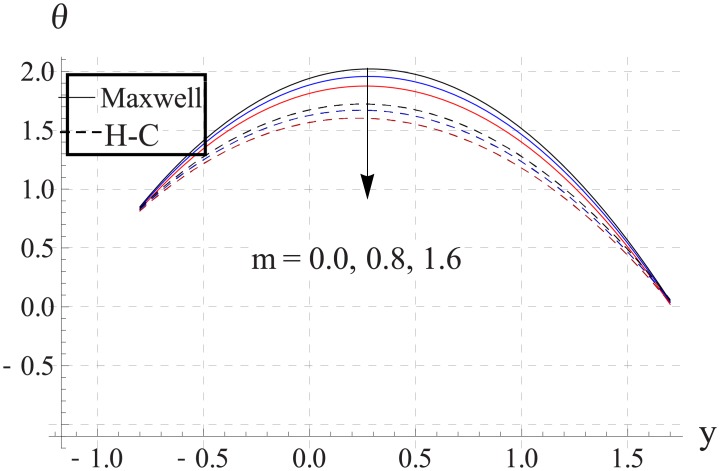
Effect of m on temperature for TiO_2_ when a = 0.7, b = 0.6, γ = π/2, d = 0.8, x = 0, η = 0.7, Br = 0.3, ϕ = 0.2, M = 2.0, Bi_1_ = 8, Bi_2_ = 10, Gr = 3.0, ε = 2.5.

**Fig 29:**
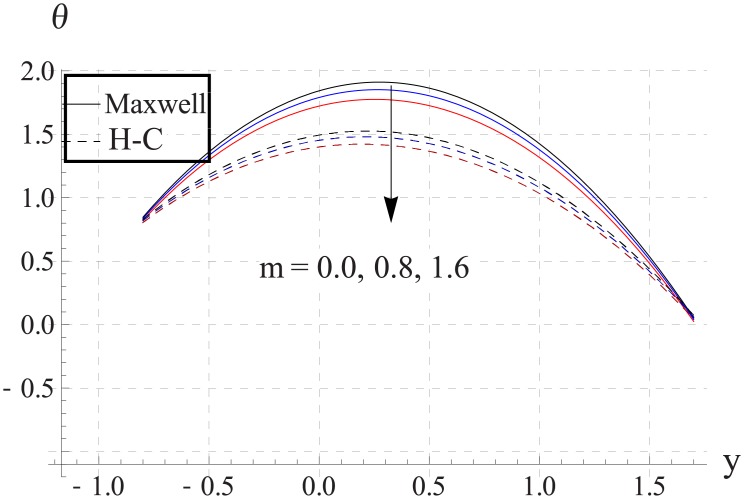
Effect of m on temperature for Al_2_O_3_ when a = 0.7, b = 0.6, γ = π/2, d = 0.8, x = 0, η = 0.7, Br = 0.3, ϕ = 0.2, M = 2.0, Bi_1_ = 8, Bi_2_ = 10, Gr = 3.0, ε = 2.5.

**Fig 30:**
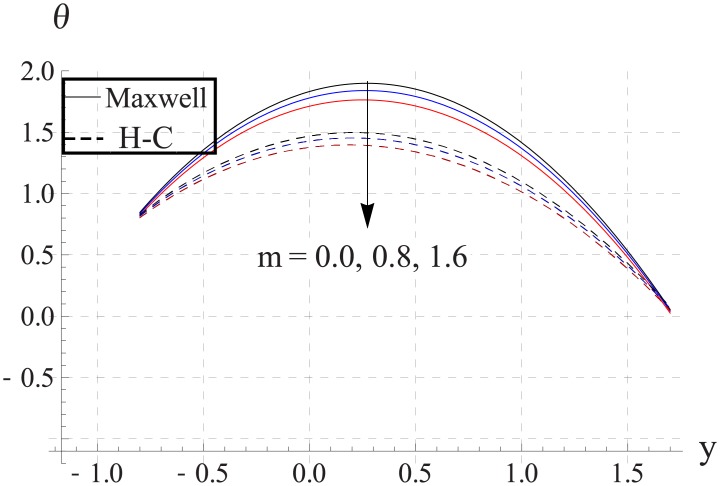
Effect of m on temperature for CuO when a = 0.7, b = 0.6, γ = π/2, d = 0.8, x = 0, η = 0.7, Br = 0.3, ϕ = 0.2, M = 2.0, Bi_1_ = 8, Bi_2_ = 10, Gr = 3.0, ε = 2.5.

**Fig 31:**
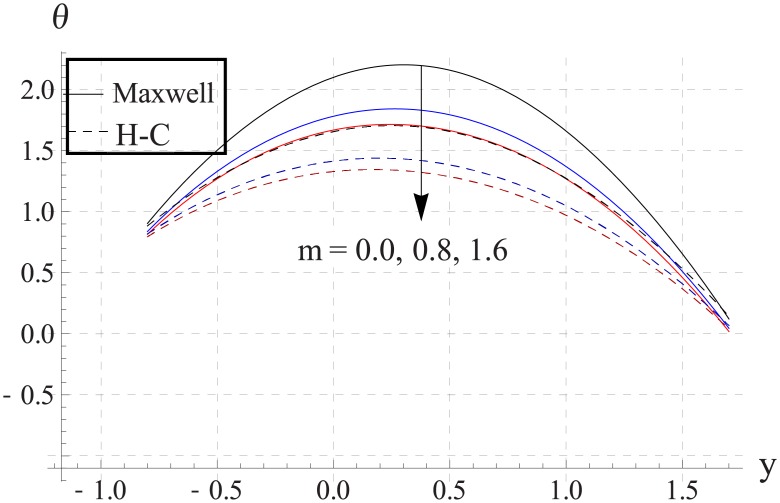
Effect of m on temperature for Cu when a = 0.7, b = 0.6, γ = π/2, d = 0.8, x = 0, η = 0.7, Br = 0.3, ϕ = 0.2, M = 2.0, Bi_1_ = 8, Bi_2_ = 10, Gr = 3.0, ε = 2.5.

**Fig 32:**
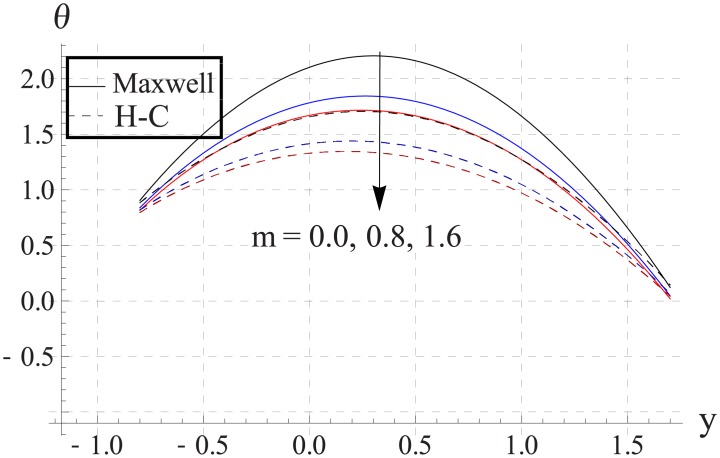
Effect of m on temperature for Ag when a = 0.7, b = 0.6, γ = π/2, d = 0.8, x = 0, η = 0.7, Br = 0.3, ϕ = 0.2, M = 2.0, Bi_1_ = 8, Bi_2_ = 10, Gr = 3.0, ε = 2.5.

Hall parameter describes opposite behavior on temperature than Hartman number (see Figs 28–32. Decrease in temperature is observed for an increase in the Hall parameter. It depicts that the Hall parameter resists the enhancement in the nanofluid temperature brought by an increase in the strength of applied magnetic field. Note that in all cases the value of Maxwell's model is greater than the Hamilton-Crosser's model. Moreover the difference between the two models is larger when we increase the thermal conductivity of the nanoparticles.

Figs 33–42 describe the impact of Biot numbers on the temperature. Increasing the values of Biot numbers reduces the temperature of the nanofluid. The values for Maxwell's model are greater in comparison to H-C's model. Furthermore the difference between the two models is larger when we increase the thermal conductivity of the nanoparticles.

**Fig 33:**
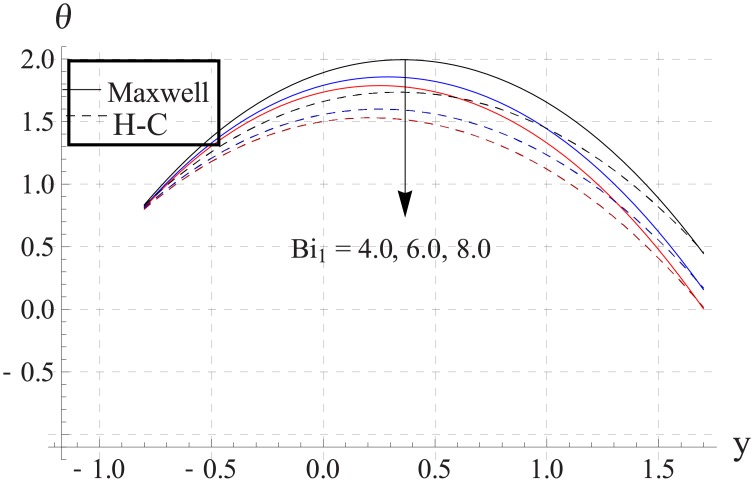
Effect of Bi_1_ on temperature for TiO_2_ when a = 0.7, b = 0.6, γ = π/2, d = 0.8, x = 0, η = 0.7, Br = 0.3, ϕ = 0.2, m = 2.0, M = 1.0, Bi_2_ = 10, Gr = 3.0, ε = 2.5.

**Fig 34:**
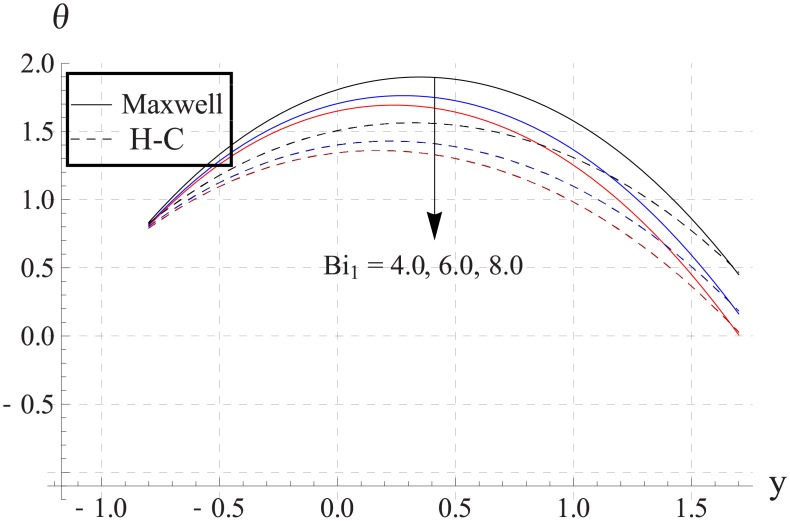
Effect of Bi_1_ on temperature for Al_2_O_3_ when a = 0.7, b = 0.6, γ = π/2, d = 0.8, x = 0, η = 0.7, Br = 0.3, ϕ = 0.2, m = 2.0, M = 1.0, Bi_2_ = 10, Gr = 3.0, ε = 2.5.

**Fig 35:**
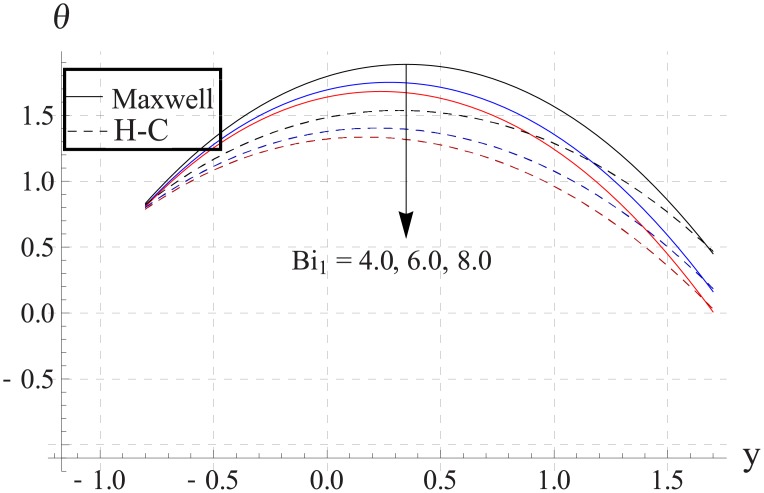
Effect of Bi_1_ on temperature for CuO when a = 0.7, b = 0.6, γ = π/2, d = 0.8, x = 0, η = 0.7, Br = 0.3, ϕ = 0.2, m = 2.0, M = 1.0, Bi_2_ = 10, Gr = 3.0, ε = 2.5.

**Fig 36:**
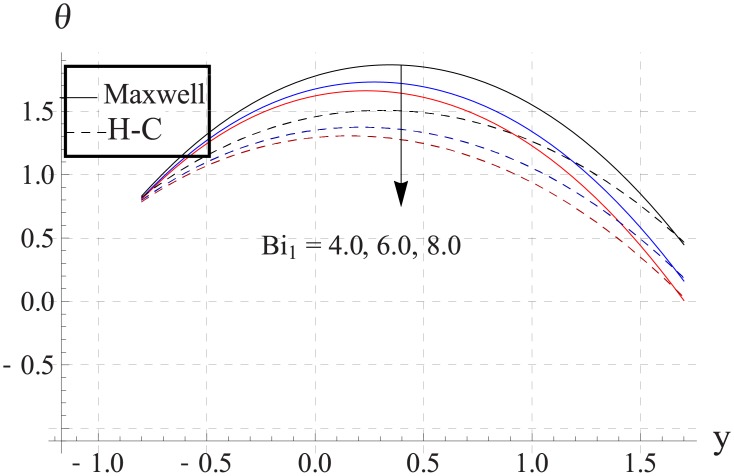
Effect of Bi_1_ on temperature for Cu when a = 0.7, b = 0.6, γ = π/2, d = 0.8, x = 0, η = 0.7, Br = 0.3, ϕ = 0.2, m = 2.0, M = 1.0, Bi_2_ = 10, Gr = 3.0, ε = 2.5.

**Fig 37:**
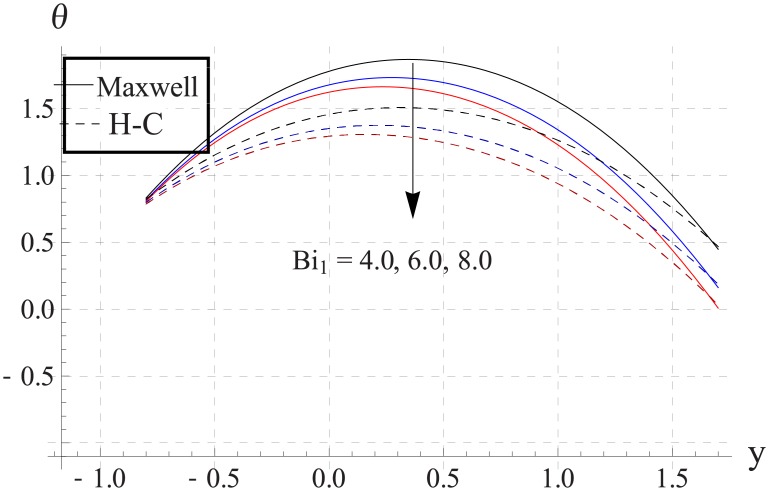
Effect of Bi_1_ on temperature for Ag when a = 0.7, b = 0.6, γ = π/2, d = 0.8, x = 0, η = 0.7, Br = 0.3, ϕ = 0.2, m = 2.0, M = 1.0, Bi_2_ = 10, Gr = 3.0, ε = 2.5.

**Fig 38:**
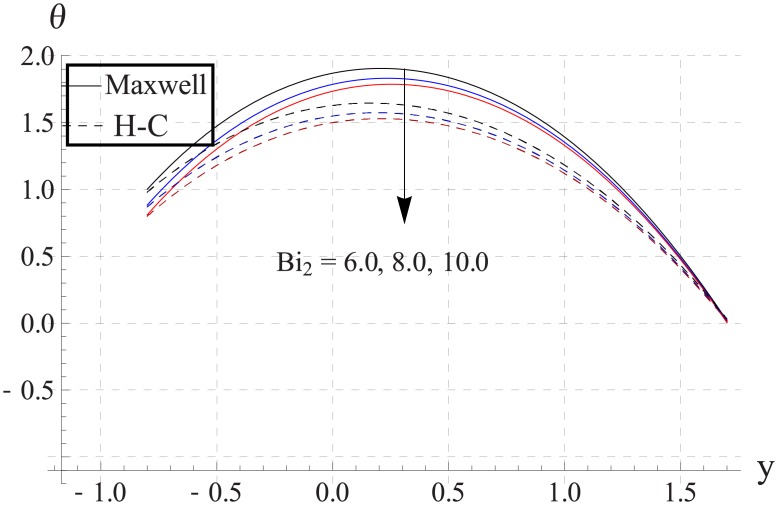
Effect of Bi_2_ on temperature for TiO_2_ when a = 0.7, b = 0.6, γ = π/2, d = 0.8, x = 0, η = 0.7, Br = 0.3, ϕ = 0.2, m = 2.0, M = 1.0, Bi_1_ = 8, Gr = 3.0, ε = 2.5.

**Fig 39:**
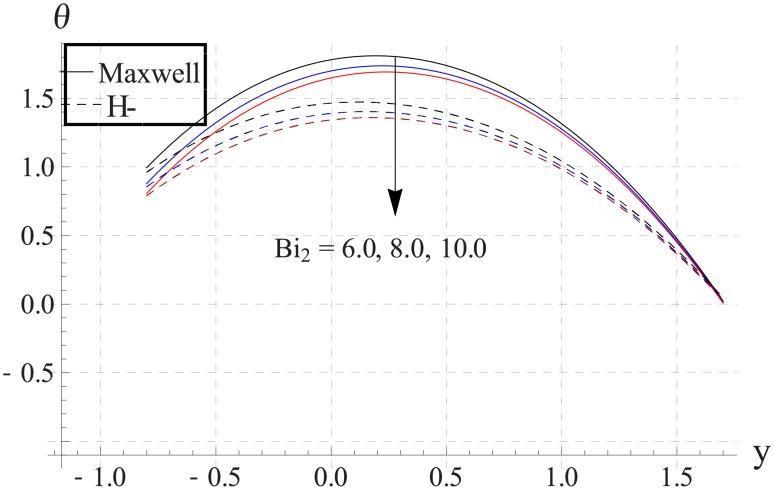
Effect of Bi_2_ on temperature for Al_2_O_3_ when a = 0.7, b = 0.6, γ = π/2, d = 0.8, x = 0, η = 0.7, Br = 0.3, ϕ = 0.2, m = 2.0, M = 1.0, Bi_1_ = 8, Gr = 3.0, ε = 2.5.

**Fig 40:**
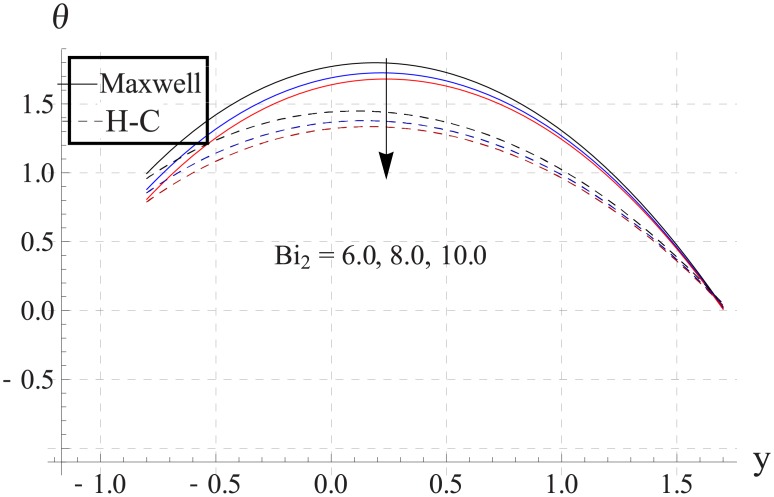
Effect of Bi_2_ on temperature for CuO when a = 0.7, b = 0.6, γ = π/2, d = 0.8, x = 0, η = 0.7, Br = 0.3, ϕ = 0.2, m = 2.0, M = 1.0, Bi_1_ = 8, Gr = 3.0, ε = 2.5.

**Fig 41:**
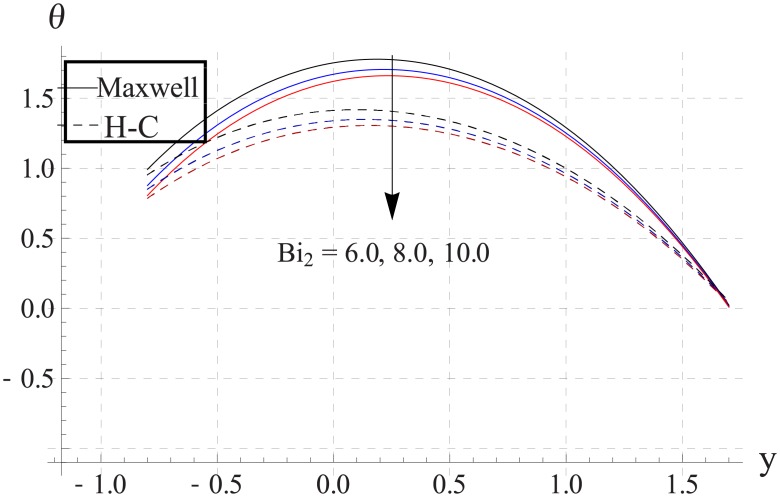
Effect of Bi_2_ on temperature for Cu when a = 0.7, b = 0.6, γ = π/2, d = 0.8, x = 0, η = 0.7, Br = 0.3, ϕ = 0.2, m = 2.0, M = 1.0, Bi_1_ = 8, Gr = 3.0, ε = 2.5.

**Fig 42:**
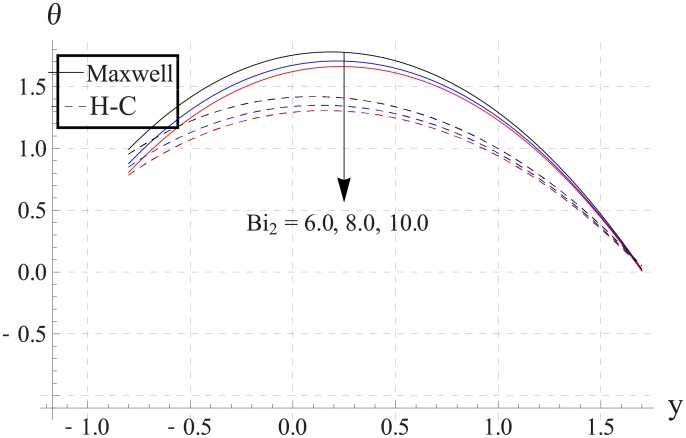
Effect of Bi_2_ on temperature for Ag when a = 0.7, b = 0.6, γ = π/2, d = 0.8, x = 0, η = 0.7, Br = 0.3, ϕ = 0.2, m = 2.0, M = 1.0, Bi_1_ = 8, Gr = 3.0, ε = 2.5.

**Fig 43:**
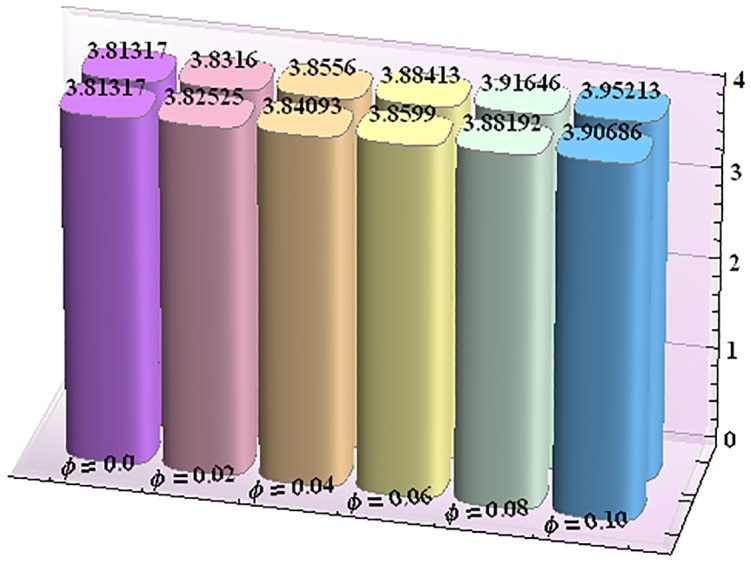
Effect of ϕ on the heat transfer rate at the wall $\left(-\frac{K_{eff}}{K_{f}}\theta^{\prime }\left(h_{1}\right)\right)$ for TiO_2_-water nanofluid when a = 0.7, b = 0.6, γ = π/2, d = 0.8, x = 0, η = 0.7, Br = 0.3, m = 0.8, M = 1.0, Bi_1_ = 8, Bi_2_ = 10, Gr = 3.0, ε = 2.5.

**Fig 44:**
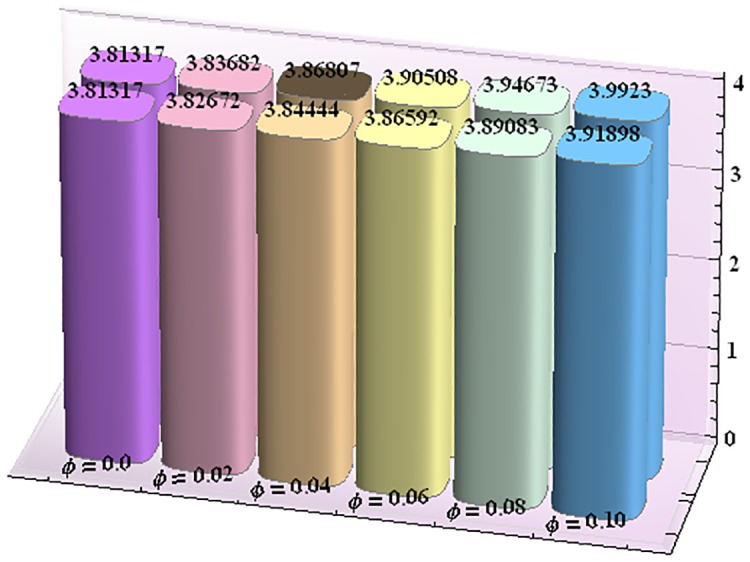
Effect of ϕ on the heat transfer rate at the wall $\left(-\frac{K_{eff}}{K_{f}}\theta^{\prime }\left(h_{1}\right)\right)$ for Al_2_O_3_-water nanofluid when a = 0.7, b = 0.6, γ = π/2, d = 0.8, x = 0, η = 0.7, Br = 0.3, m = 0.8, M = 1.0, Bi_1_ = 8, Bi_2_ = 10, Gr = 3.0, ε = 2.5.

**Fig 45:**
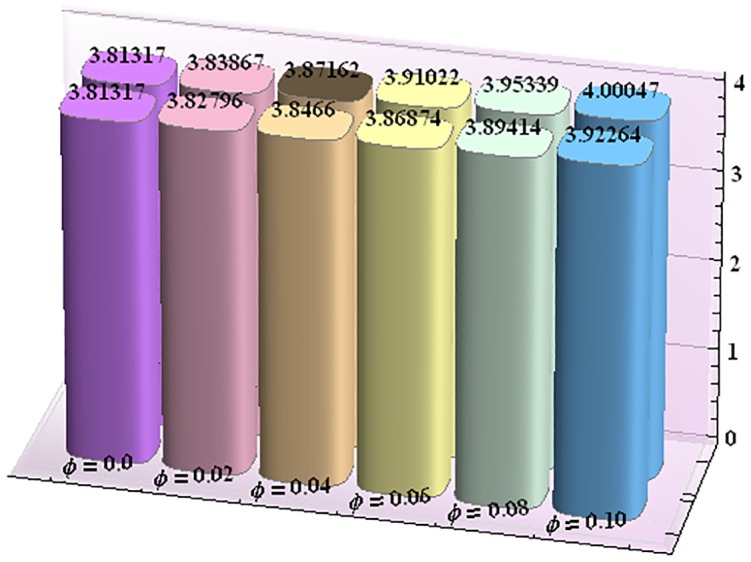
Effect of ϕ on the heat transfer rate at the wall $\left(-\frac{K_{eff}}{K_{f}}\theta^{\prime }\left(h_{1}\right)\right)$ for CuO-water nanofluid when a = 0.7, b = 0.6, γ = π/2, d = 0.8, x = 0, η = 0.7, Br = 0.3, m = 0.8, M = 1.0, Bi_1_ = 8, Bi_2_ = 10, Gr = 3.0, ε = 2.5.

**Fig 46:**
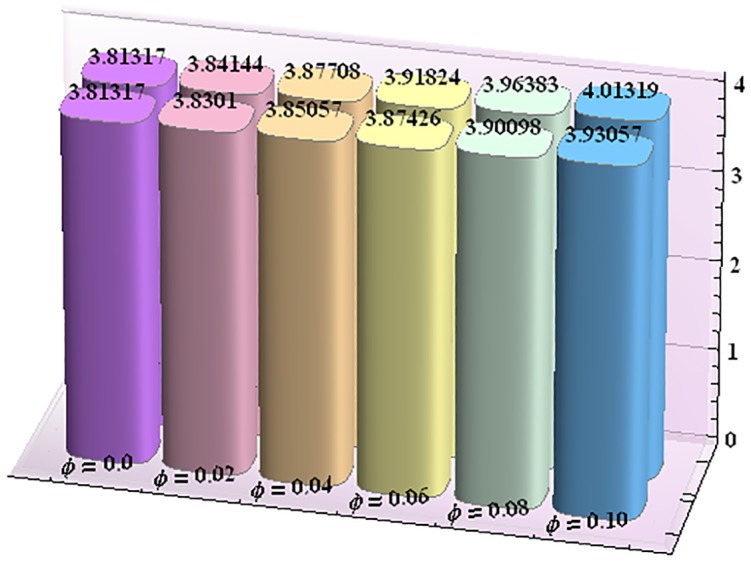
Effect of ϕ on the heat transfer rate at the wall $\left(-\frac{K_{eff}}{K_{f}}\theta^{\prime }\left(h_{1}\right)\right)$ for Cu-water nanofluid when a = 0.7, b = 0.6, γ = π/2, d = 0.8, x = 0, η = 0.7, Br = 0.3, m = 0.8, M = 1.0, Bi_1_ = 8, Bi_2_ = 10, Gr = 3.0, ε = 2.5.

**Fig 47:**
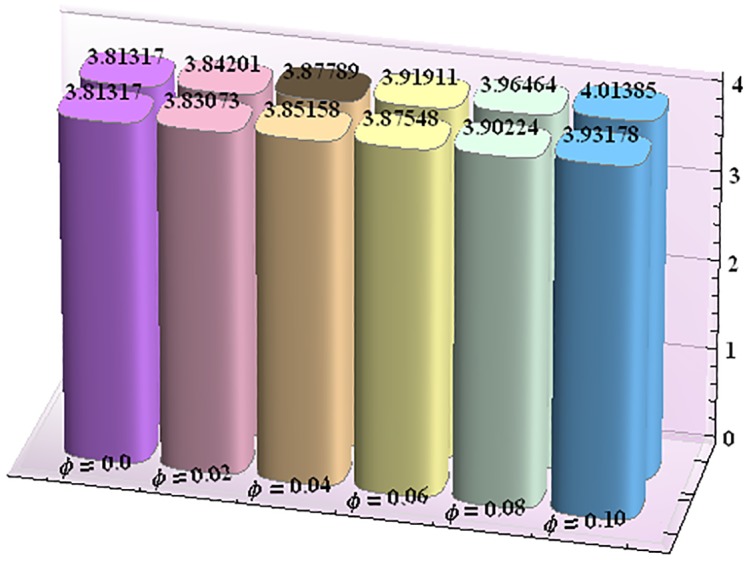
Effect of ϕ on the heat transfer rate at the wall $\left(-\frac{K_{eff}}{K_{f}}\theta^{\prime }\left(h_{1}\right)\right)$ for Ag-water nanofluid when a = 0.7, b = 0.6, γ = π/2, d = 0.8, x = 0, η = 0.7, Br = 0.3, m = 0.8, M = 1.0, Bi_1_ = 8, Bi_2_ = 10, Gr = 3.0, ε = 2.5.

Figs 43–47 portray the impact of nanoparticle volume fraction on the rate of heat transfer at the wall. Study discloses the fact that the heat transfer rate at the wall is increasing function of nanoparticle volume fraction ϕ. For H-C's model the heat transfer rate is higher than the Maxwell's model. Moreover rate of heat transfer is increased by enhancing the thermal conductivity of nanoparticles. Also by an increase in volume fraction the gap between the values of two models widens.

## Conclusions

Here we analyzed the MHD mixed convection peristaltic transport of water-based nanofluids by employing convective conditions on the channel boundaries. The following observations are worth mentioning.

- By nanoparticle volume fraction, both the axial velocity and temperature are decreased. Increasing the nanoparticles the difference between the values of two models increases. Such increase is larger for metallic particles.
- By increasing the strength of magnetic field the velocity of nanofluid decreases. Larger strength of magnetic field increased the difference between the two models. For metallic particles this gap widens than metallic oxides.
- By increasing the strength of magnetic field the temperature of nanofluid increases in view of Joule heating. Increasing strength of magnetic field enhanced the difference between the two models. For metallic particles this gap is more than metallic oxides.
- Hartman number and Hall parameter shows opposite behavior on the velocity and temperature.
- With an increase in the Biot number the temperature of the nanofluid decreases. For the nanoparticles with high thermal conductivity the difference between the values of two models is large.
- Heat transfer rate at the wall enhances with increasing nanoparticle volume fraction. Moreover such increase is larger for cylindrical nanoparticles than the spherical ones.
